# Bacterial polyextremotolerant bioemulsifiers from arid soils improve water retention capacity and humidity uptake in sandy soil

**DOI:** 10.1186/s12934-018-0934-7

**Published:** 2018-05-31

**Authors:** Noura Raddadi, Lucia Giacomucci, Ramona Marasco, Daniele Daffonchio, Ameur Cherif, Fabio Fava

**Affiliations:** 1 Department of Civil, Chemical, Environmental and Materials Engineering (DICAM), Alma Mater Studiorum University of Bologna, via Terracini 28, 40131 Bologna, Italy; 2 Biological and Environmental Sciences and Engineering Division, King Abdullah University of Science and Technology (KAUST), Thuwal, 23955-6900 Saudi Arabia; 3 LR Biotechnology and Bio-Geo Resources Valorization, Higher Institute for Biotechnology, Biotechpole Sidi Thabet, University of Manouba, 2020 Ariana, Tunisia

**Keywords:** Polyextremotolerant bioemulsifiers, Desert sandy soil, Water retention, Humidity uptake, Water stress

## Abstract

**Background:**

Water stress is a critical issue for plant growth in arid sandy soils. Here, we aimed to select bacteria producing polyextremotolerant surface-active compounds capable of improving water retention and humidity uptake in sandy soils.

**Results:**

From Tunisian desert and saline systems, we selected eleven isolates able to highly emulsify different organic solvents. The bioemulsifying activities were stable with 30% NaCl, at 4 and 120 °C and in a pH range 4–12. Applications to a sandy soil of the partially purified surface-active compounds improved soil water retention up to 314.3% compared to untreated soil. Similarly, after 36 h of incubation, the humidity uptake rate of treated sandy soil was up to 607.7% higher than untreated controls.

**Conclusions:**

Overall, results revealed that polyextremotolerant bioemulsifiers of bacteria from arid and desert soils represent potential sources to develop new natural soil-wetting agents for improving water retention in arid soils.

**Electronic supplementary material:**

The online version of this article (10.1186/s12934-018-0934-7) contains supplementary material, which is available to authorized users.

## Background

Biosurfactants (BS) are amphipathic compounds produced by a variety of microorganisms. They can be low-molecular weight, generally glycolipids or lipopeptides, or high-molecular weight compounds, which are mainly lipopolysaccharides, lipoproteins, or a combination of both. The high-molecular weight BS, also called bioemulsifiers (BE), are capable of producing stable emulsions, but do not always determine decreases of surface or interfacial tensions [1]. There is a growing interest in microbial biosurfactants owing to several advantages over conventional surfactants, including biodegradability, low toxicity and production from renewable substrates [2–4].

BE and BS are used in agriculture for several applications ranging from the improvement of the quality of polluted soils, to the control of plant pathogens or for favoring plant–microbe interactions [5]. The potential of exopolysaccharides (EPS) with bioemulsifying properties have been considered for the promotion of plant growth by bacterial producers [6, 7] or for their effect on the hydrological behavior of biological soil crusts [8]. Application of surfactants to soil has been included among twelve strategies for the remediation of soil water repellency [9], even though the effect of biosurfactants on soil water repellency are controversial. Some studies have reported that fungi may produce hydrophobins that favor the development of water repellency [10].

One aspect that, to our knowledge, has not been considered in the literature on BS/BE is their potential for improving soil water retention [8], especially in arid and desert sandy soils that have limited content of organic matter and experience extreme environmental conditions of limited water supply and nutrient content, high temperatures, irradiation and evaporation rates and high salinity [11, 12].

We hypothesize that, by living under extreme conditions, bacteria in arid and desert soils should have evolved capacities and strategies that allow an efficient use of water, including production of BS/BE highly stable under extreme conditions of temperature and irradiation and that can facilitate access/retention of low-available water and nutrients. On the plant leaf surface that experiences analogous extremes such as those in desert sandy soils [13], epiphytic bacteria have been reported to exploit biosurfactants to increase wettability of the leaf and to enhance nutrients diffusion through the wax cuticle [14]. Similarly, bacteria inhabiting arid environments and especially sandy soils could take advantage from BS/BE to access limited nutrient supplies and withstand fluctuations in moisture availability in a water-deficient environment.

In this study, we have screened a collection of bacteria from arid Tunisian soils including (i) an inland saline system and sand from the Sahara desert and (ii) an arid vineyard in the North of the country, for BS/BE activities under different conditions. We have also tested a subgroup of selected BS/BE bacterial isolates for their potential to improve water retention and humidity uptake in sandy soils.

## Methods

### Bacterial strains

The 24 bacterial isolates used in this study include 23 strains obtained from sediment samples recovered from the inland saline system Chott El Fejej and desert sand from Douz in the South of Tunisia (Additional file 1: Table S1); and one (V3E1) isolated from the root system of grapevine plants growing in an arid soil of Northern Tunisia (Mornag area). The strains were identified based on partial 16S rRNA gene sequences (database accession numbers MG594617-MG594638 and MG637030).

### Screening for biosurfactant/bioemulsifier production

Cells were inoculated into glucose mineral salts medium (GMSM) (g/L: 20.0 glucose; 0.7 KH_2_PO_4_; 0.9 Na_2_HPO_4_; 2.0 NaNO_3_; 0.4 MgSO_4_·7H_2_O; 0.1 CaCl_2_·2H_2_O; 2 mL of trace elements [per liter, 2.0 g FeSO_4_·7H_2_O, 1.5 g MnSO_4_·H_2_O, 0.6 g (NH_4_)_6_Mo_7_O_24_·4H_2_O]; pH = 6.72). The flasks were incubated at 30 °C on a rotary shaker (150 rpm). The surface activity of cell-free supernatants was tested on culture samples every 24 h for a period of up to 4 days. The cell-free culture supernatants recovered by centrifugation (10,000 rpm, 10 min, 4 °C) followed by filter-sterilization (0.22 µm) were used for evaluation of the surface activity by determining the emulsification index and the drop collapse activity as well as by measuring the interfacial surface tension (IFT).

The emulsification activity (EA) of the supernatant was determined as follows. Two milliliters of culture supernatant and an equal volume of the tested organic solvent (*n*-hexane, *n*-hexadecane or toluene) were placed in a test tube that was vortexed at high speed for 2 min and then allowed to settle for 24 h. The emulsification index (EI_24_%) was estimated as the height of the emulsion layer, divided by the total height, multiplied by 100.

The surface qualitative drop collapse activity test was carried out as follows: 40 µL of the cell free supernatant was aliquoted as a droplet onto Parafilm^®^ (Parafilm M, Germany); the flattening and the spreading of the droplet on the Parafilm^®^ surface was followed over 10 min and recorded by visual inspection. The assay was scored negative or positive if the drop remained beaded or collapsed, respectively.

The IFT of the cell-free culture supernatant was measured with a Drop Shape Analyzer—DSA30 (KRUSS GmbH, Germany) working in the pendant drop mode. The drops were produced by a syringe equipped with a 2.098 mm needle diameter, were left to equilibrate close to the rupture point and IFT values (mN/m) were calculated by the instrument software using the Young–Laplace equation:

$$\Delta p\, = \,\sigma *\left( {{ 1\mathord{\left/ {\vphantom { 1{r1}}} \right. \kern-0pt} {r1}}\, + \,{1 \mathord{\left/ {\vphantom {1 {r2}}} \right. \kern-0pt} {r2}}} \right);$$

where ∆*p* is the differential pressure between the inside and outside of the drop, σ is the IFT value and *r*1 and *r*2 are the main radii of the drop curvature. Measurements were performed at room temperature, at least in triplicates. A Tween 80 solution at a final concentration of 0.5%_w/v_ (in water), was used as positive control while deionized water and non-inoculated growth medium with 2%_w/v_ glucose were used as negative controls. All screening assays were performed in duplicate as independent experiments.

### Stability of bioemulsifiers

Emulsion stability studies were performed using cell-free culture supernatants. The stability of the bioemulsifiers activity was determined by investigating the effect of varying NaCl concentration, temperature or pH on the EA. In order to assess the effect of salinity on the bioemulsifier activity, culture supernatants were supplemented with different NaCl concentrations (8–30%_w/v_) and the emulsifying activity was measured as described above. To evaluate the stability of the bioemulsifier at different temperatures, culture supernatants were subjected to cooling (4 °C, 2 h), heating (55 °C, 2 h) or autoclaving (121 °C for 20 min) followed by cooling to room temperature before performing the EI assay. The measured emulsification indexes were compared to the corresponding values obtained using culture supernatants not subjected to the different treatments. The EA of hexadecane was not evaluated at 4 °C since the melting point of the solvent was of 18 °C and hence at lower temperature the emulsion was subjected to solidification. The pH stability was studied by adjusting the cell-free culture supernatants to different pH values (4–12) using HCl or NaOH solutions, and then the EA was measured as previously described. Furthermore, the stability of the emulsions produced under the different conditions was monitored for up to 30 months.

All the experiments were carried out in duplicate. The results were reported as residual emulsification activity (REA) percentage (%) expressed as follows:

$${\text{REA}}\;\left( \% \right)\, = \,{{{\text{EI}}_{\text{t}} } \mathord{\left/ {\vphantom {{{\text{EI}}_{\text{t}} } {{\text{EI}}_{24} }}} \right. \kern-0pt} {{\text{EI}}_{24} }}*100;$$

where EI_t_ and EI_24_ are the EI (%) values at incubation time t and after 24 h, respectively; and compared with those obtained with the positive control.

### Water retention and moisture uptake assays by a sandy soil

For BS/BE production, selected bacterial strains were grown in GMSM and incubated at 30 °C. Cells were removed by centrifugation (8000 rpm, 10 min, 4 °C) and the collected supernatant was acidified with 6 N hydrochloric acid solution to pH 2.0 ± 0.3. The precipitate which contained BS/BE was allowed to settle at 4 °C overnight. The precipitated BS/BE were collected by centrifugation (12,000 rpm, 20 min, 4 °C) and resuspended in sterile tap water at a final concentration of 25 g/L. Triton X-100 applied at a final concentration corresponding to its CMC (0.14 g/L) and sterilized tap water were used as controls. Before using in the experiments, the sandy soil sample was oven-dried (105 °C, 4 h) and subjected to sterilization by tyndallization followed by drying (55 °C, overnight) and acclimated at 30 °C for 24 h.

The water retention assay was performed as follows: 10 g of sandy soil were placed in a glass Petri dish in duplicate and subjected to wetting with 10 ml of a solution of BS/BE to be tested or tap water, followed by drying at 30 °C until a constant weight was achieved. The samples have been thoroughly mixed manually and then subjected to two cycles of irrigation with 2.5 mL sterilized tap water and drying until constant weight was achieved. During the first drying cycle, each sample was weighed immediately after irrigation with tap water and every 60 min up to 12.5 h of incubation and then each 12 h until constant weight was achieved. Monitoring of the samples weight after the second irrigation was performed at the time of irrigation, after 12.5 h of incubation and then every 60 min until complete drying.

The moisture uptake assay was performed by incubating the Petri dishes containing 10 g of dried sandy soil samples in a sealed desiccator, in which the dishes were placed on the platform and the space under the platform was filled with tap water instead of desiccant in order to create a high (100%) relative humidity in the environment. After 36 h of incubation at constant temperature (30 °C), the samples were weighed in order to evaluate their weight increase due to humidity uptake.

### Statistical analyses

Data related to the evaluation of bioemulsifiers stability under stressful conditions and to water retention and moisture uptake assays were statistically assessed using the analysis of variance (ANOVA) via MATLAB software (Version R2017a, The MathWorks Inc, Natick, USA). The statistical analyses aimed to highlight the statistically significant differences among EI_24_% values obtained under standard and stressful conditions, and among the amounts of retained water and uptaken humidity by sandy soil samples treated with the bioemulsifiers solutions, Triton X-100 or tap water. The significance of the data was determined by *Tukey* honestly significant different test. Statistically significant results were depicted by *p* values < 0.05.

## Results and discussion

### Screening of surface-active strains and their identification

Among the 23 isolates screened (Additional file1: Table S1, Additional file2: Figure S1), 9 were capable to significantly emulsify (EI_24_% > 45%) at least one of the solvents tested (Table 1). On the basis of the partial 16S rRNA gene sequences, all isolates had between 99 and 100% sequence similarities with their closest relative type strain in the databases (Table 1). *Bacillus mojavensis* IFO15718 isolated from Mojave desert [15, 16] was the closest relative species of the R4p, R43 and R39 strains, while *Bacillus endophyticus* strain 2DT isolated from the inner tissues of cotton plants [17] was the closest relative of L45, L97b and L37 isolates. The partial 16S rRNA gene sequence from strain N3 showed 99% homology with different *B. subtilis* including the subsp. *spizizenii *TU-B-10 isolated from soil collected near Nefta, Tunisia [18]. *Bacillus licheniformis* strain DSM 13 was the closest relative of L98 strain, and *Bacillus frigoritolerans* DSM 8801 isolated from arid soil in Morocco was that of R55 and R40 strains. The last isolate among those obtained from chott and that is positive for BS/BE production was previously classified as *Paenibacillus tarimensis* (strain L88) [19], while the grapevine rhizosphere isolate V3E1was assigned to *Rhizobium* sp..

**Table 1:** List of BS/BE producers obtained from the screening of 23 desert bacterial isolates grown on MSM with 2%_w/v_ glucose as carbon source. Emulsification index (EI_24_%) and interfacial surface tension (IFT) values are expressed and mean value ± SD of two and three replicates, respectively, while drop collapse results as positive (+) or negative (−)

Isolate ID	16SrDNA Accession No	Closest type strains (GenBank Accession No)	16S rDNA identity (%)	Drop collapse	IFT (mN/m)	EI24 (%)		
						Toluene	Hexane	Hexadecane
*Bacillus* sp. L37	MG594627	*Bacillus endophyticus* (NR_025122)	99	+	69.28 ± 0.31	57.63 ± 4.24	56.07 ± 2.18	61.11 ± 1.92
*Bacillus* sp. L45	MG594628	*Bacillus endophyticus* (NR_025122)	99	−	70.00 ± 0.47	52.53 ± 1.14	59.67 ± 1.48	53.33 ± 9.43
*Paenibacillus tarimensis* L88	KF111690	*Paenibacillus tarimensis* (NR_044102)	99	−	74.45 ± 0.16	44.64 ± 2.06	50.93 ± 1.07	48.32 ± 6.27
*Bacillus* sp. L97b	MG594634	*Bacillus endophyticus* (NR_025122)	99	−	72.48 ± 0.36	57.64 ± 3.67	53.11 ± 5.13	47.36 ± 8.45
*Bacillus* sp. L98	MG594631	*Bacillus licheniformis* (NR_118996)	99	+	55.07 ± 0.26	48.82 ± 6.06	50.83 ± 2.18	46.43 ± 5.05
*Bacillus* sp. N3	MG594629	*Bacillus subtilis* subsp. *spizizenii *(NR_112686)	99	+	36.86 ± 1.11	50.81 ± 2.17	34.84 ± 3.21	51.67 ± 7.07
*Bacillus* sp. R4p	MG594635	*Bacillus mojavensis* (NR_112725)	99	+	28.99 ± 0.35	55.67 ± 6.95	44.91 ± 4.96	28.57 ± 0.00
*Bacillus* sp. R39	MG594626	*Bacillus mojavensis* (NR_112725)	99	+	28.36 ± 0.63	11.15 ± 0.00	5.30 ± 2.26	0.00 ± 0.00
*Bacillus* sp. R40	MG594633	*Bacillus frigoritolerans* (NR_115064)	99	−	70.25 ± 0.46	52.12 ± 7.54	44.90 ± 4.52	58.33 ± 2.36
*Bacillus* sp. R43	MG594630	*Bacillus mojavensis* (NR_112725)	99	+	28.57 ± 0.76	26.67 ± 1.33	32.69 ± 6.78	11.51 ± 2.59
*Bacillus* sp. R55	MG594632	*Bacillus frigoritolerans* (NR_115064)	99	−	71.10 ± 0.85	59.98 ± 2.54	49.20 ± 2.92	53.77 ± 8.08

The eleven desert/chott isolates capable of producing BS/BE were spore-formers, a group of bacteria well adapted to the arid conditions of the desert primarily for their capacity to produce spores that are resistant to heating, desiccation and irradiation [20]. Several of the strains were affiliated to species previously isolated from soil and sand from other desert ecosystems, such as *Bacillus mojavensis* [15] and *Paenibacillus tarimensis* [21].

Even though it should be confirmed by a larger range of isolates and ecosystems, it can be noted that comparing the bacterial BS/BE producers with the initial collection of isolates (Additional file1: Table S1), almost all of them (10 out of 11) belong to the genus *Bacillus*. This observation suggests that that BS/BE activities are important features in the *Bacillus* genus for the adaptation to arid conditions.

The maximum emulsifying activity was recorded between 24 and 96 h of incubation. The significant highest emulsification index was observed using hexadecane as solvent (61.11 ± 1.92% by *Bacillus endophyticus* L37; Table 1). Cell-free culture supernatants of four isolates (*Bacillus* sp. *isolate* N3, *Bacillus* sp. isolates R4p, R39 and R43) significantly decreased the medium surface tension from 74.66 ± 0.21 mN/m, with a lowest value of 28.36 ± 0.63 mN/m (Table 1). These results suggest that under these experimental conditions, most of the isolates (8 out of 11) produced BE since their culture supernatants did not exhibit a remarkable surface tension reduction but were able to highly emulsify the tested organic solvents. Indeed, the formation of emulsions is typical of BE while BS are the compounds that are able to significantly reduce the surface tension of aqueous media to around 40 mN/m [14, 22].

*Bacillus* spp. are well known BS/BE producers and their applications were related to hydrocarbon (crude oil) degradation and microbial enhanced oil recovery [23–25]. Among others, *B. endophyticus* isolate TSH42 was reported to produce surfactin, fengycin and iturin lipopeptides that exhibit fungal biocontrol activity [26]; however their ability to reduce the growth medium surface tension or to emulsify organic solvents has not been evaluated previously. *B. mojavensis* has been previously studied for lipopeptide biosurfactant production [27] but not for bioemulsification activities. *B. licheniformis* was the only one previously characterized for its production of lipopeptide biosurfactants capable to reduce the medium surface tension to 28 mN/m [28] and to determine bioemulsification activity [24, 25]. Interestingly, while *Paenibacillus* sp. isolates have been previously studied for their BS/BE production and activity [29–32], no data regarding *B. frigoritolerans* were available in literature.

### Stability of bioemulsifiers

**Fig. 1:**
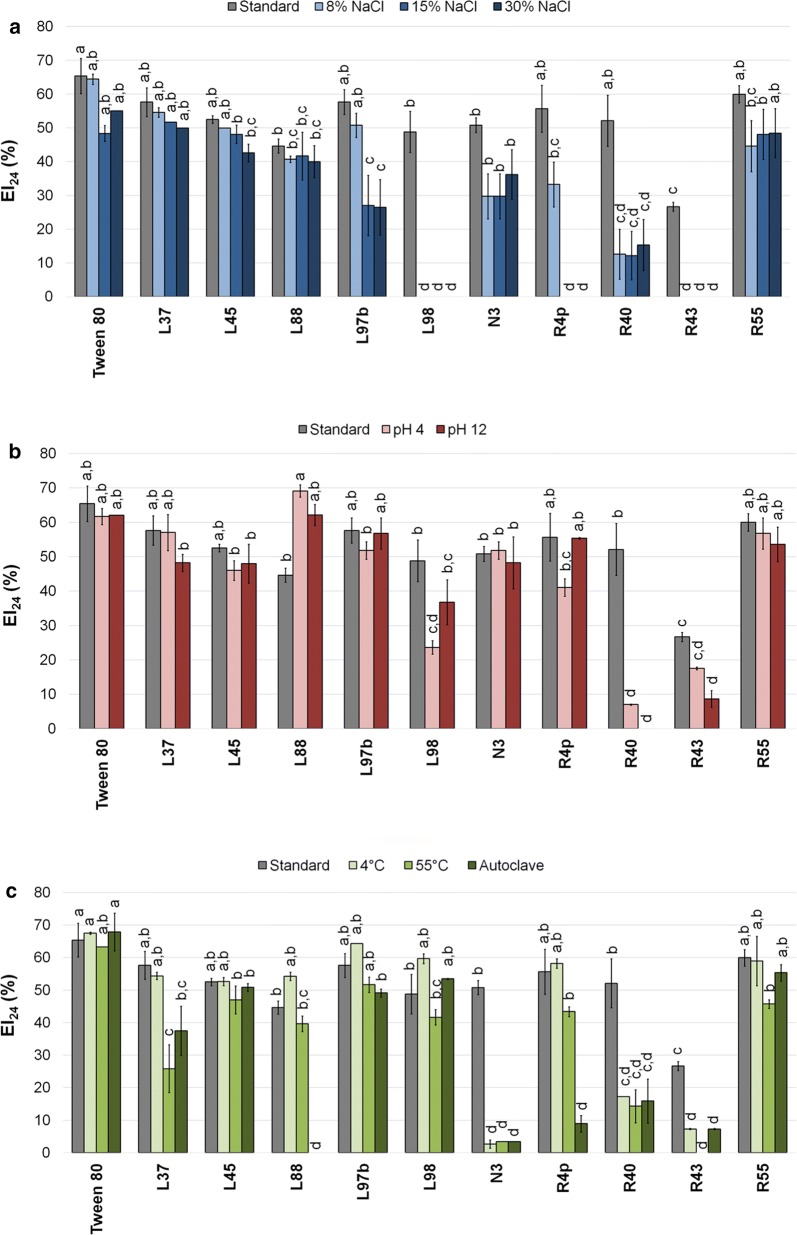
EI_24_ (%) of toluene recorded under standard and stressful conditions. EI of toluene under low water activity (ANOVA: p < 0.0001, F = 43.86, df = 43) (**a**), extremes of pH (ANOVA: p < 0.0001, F = 35.89, df = 32) (**b**) and different temperatures (ANOVA: p < 0.0001, F = 64.15, df = 43) (**c**). Results of post hoc analyses of each condition were represented as letters: different letters correspond to statistically different EI_24_ (%) results (α = 0.05)

**Fig. 2:**
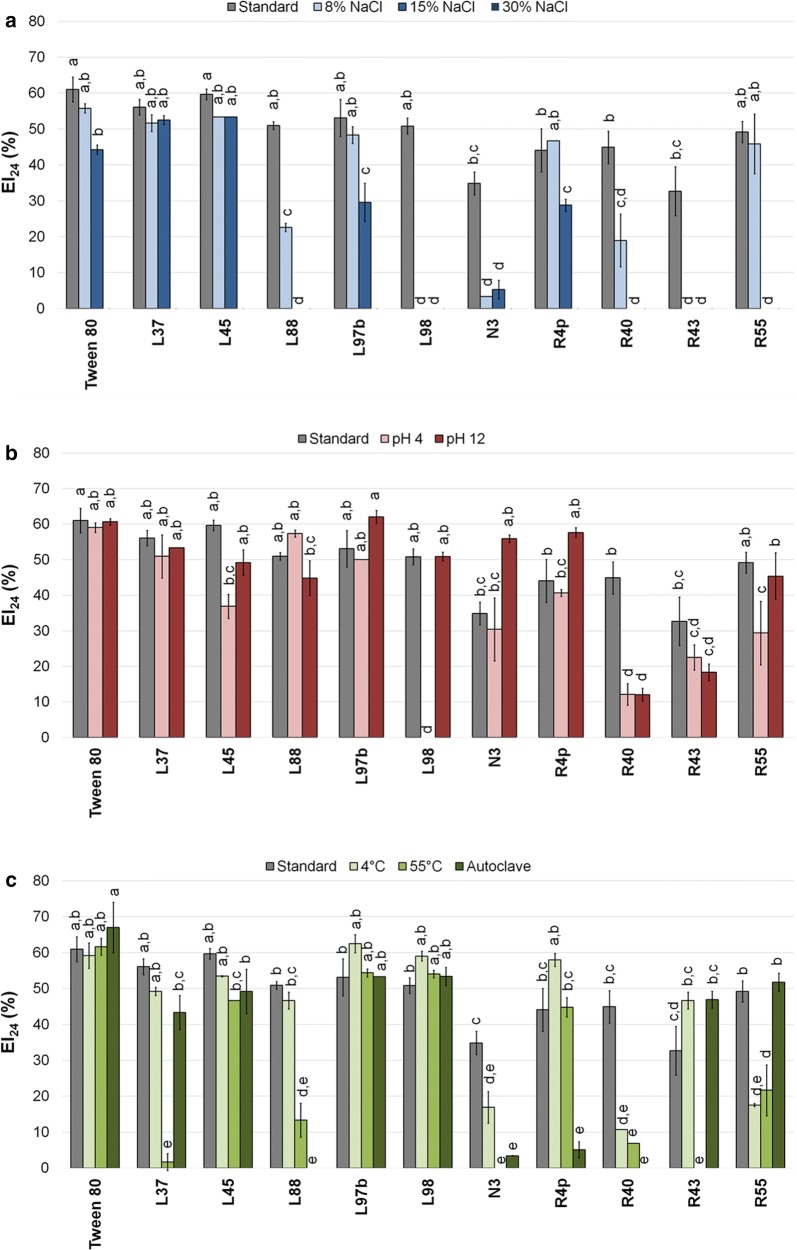
EI_24_ (%) of hexane recorded under standard and stressful conditions. EI of hexane under low water activity (ANOVA: p < 0.0001, F = 95.13, df = 32) (**a**), extremes of pH (ANOVA: p < 0.0001, F = 38.18, df = 32) (**b**) and different temperatures (ANOVA: p < 0.0001, F = 86.43, df = 43) (**c**). Results of post hoc analyses of each condition were represented as letters: different letters correspond to statistically different EI_24_ (%) results (α = 0.05)

**Fig. 3:**
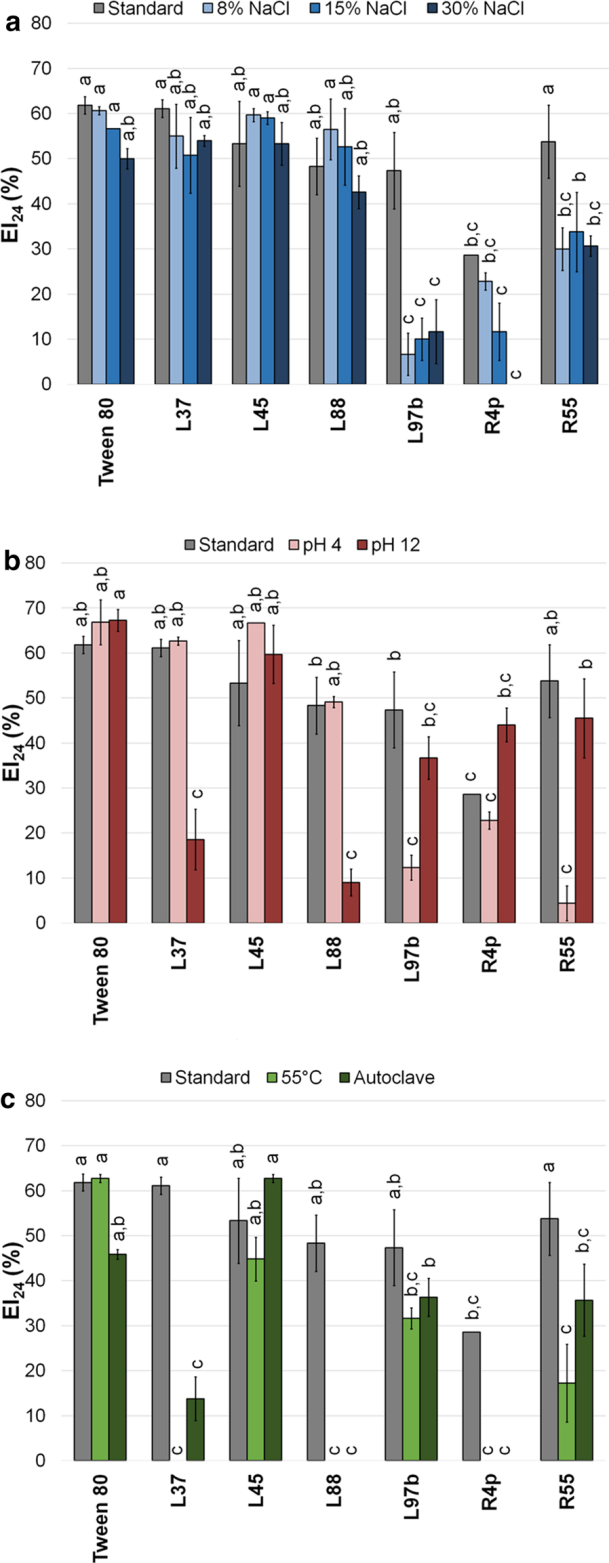
EI_24_ (%) of hexadecane recorded under standard and stressful conditions. EI of hexadecane under different salt concentrations (ANOVA: p < 0.0001, F = 28.26, df = 27) (**a**), extremes of pH (ANOVA: p < 0.0001, F = 34.44, df = 20) (**b**) and temperature (ANOVA: p < 0.0001, F = 53.73, df = 20) (**c**). Results of post hoc analyses of each condition were represented as letters: different letters correspond to statistically different EI_24_ (%) results (α = 0.05)

Based on BS/BE activities, ten bioemulsifying isolates were selected and the stability of their EA was evaluated under different conditions. The activity of the crude BEs was evaluated from cell-free culture supernatants after exposure to low water activity conditions as well as to extremes of pH or temperature (Figs. 1, 2, 3). In presence of toluene under standard conditions, six of the isolates (L37, L45, L97b, R4p, R40 and R55) did not exhibit a statistically significant decrease of their BE activity compared to the positive control (Tween 80, Fig. 1a), while EI_24_ (%) of the remaining isolates (L88, L98, N3 and R43) were significantly reduced. Different trends were observed when salt stresses were applied to the solution. With the increase of NaCl concentration (up to 30%), only five isolates (L37, L45, L88, N3 and R55) showed EI_24_ values statistically comparable to those obtained under standard conditions, suggesting how stress can strongly affect the activity of bacterial BEs. Remarkably, while the EA_24_ was significantly reduced for the strains R40 and L97b, it was completely lost for R4p, L98 and R43 (Fig. 1a).

When the stress applied was determined by extreme pH (4 and 12), a different scenario was observed (Fig. 1b). Under these conditions, no significant changes in EA_24_ values were reported for L45, L97b, R55, N3, R4p and L37, as well as for the positive control (Tween 80), compared to standard conditions. The toluene EA by culture supernatant of isolate *P. tarimensis* L88 was significantly enhanced at pH 4, while remained statistically equivalent to standard conditions at pH 12. On the contrary, a significant EA_24_ reduction/loss was recorded for isolates R40 (pH 4 and pH 12), R43 (pH 12) and L98 (pH 4) (Fig. 1b). With the exception of isolates N3, R43 and R40, all the remaining cell-free culture supernatants retained EA_24_ at low temperature (4 °C), which were statistically identical to that of Tween 80 (Fig. 1c). When the temperature was increased to 55 °C, isolates L97b, L88, L98, R4p, R55 and L45 showed EA_24_ comparable to the corresponding standard conditions and to Tween 80, while a reduction/loss of activity was recorded for strains L37, R40, N3 and R43 (Fig. 1c). After autoclaving, the cell-free culture supernatants of the tested isolates retained an EA_24_ unchanged compared to their corresponding activities under standard conditions; except for strains N3, R40, L88, R4p and R43 (Fig. 1c).

Bacterial BEs acted in a completely different way when hexane was used as solvent (Fig. 2a–c). In the presence of salt stress (15%), a significant reduction of bacterial BEs performances was observed, except for isolates L37 and L45 that showed EI_24_ values statistically comparable to the corresponding activities under standard conditions, as well as to that of Tween 80 with 15% NaCl. Reaching the maximum salt stress tested (30%), a total absence of activity was recorded for all the culture supernatants as well as for Tween 80 (Fig. 2a). In the presence of a lower NaCl concentration (8%) the hexane EA_24_ was maintained statistically unchanged compared to the standard conditions for Tween 80 and strains L37, L45, L97b, R4p and R55; while it was completely lost for R43 and L98 and significantly reduced for the remaining isolates (L88, N3, R40). Considering pH as stressful condition, under acidic conditions EA_24_ was significantly reduced for isolates R40 and R55 with a complete loss for strain L98 at pH 4 (Fig. 2b). In general, alkaline pH did not affect EA_24_ with the exception of the strain R40 (Fig. 2b). Looking to the temperature, also in this case for some strains the autoclaving procedure resulted in a reduction (N3, R4p) or complete loss (L88 and R40) of the EA_24_. The incubation at 55 °C resulted in a significant reduction of EA_24_ for the isolates L37, L88, R40 and R55 and a complete loss for N3 and R43, while the activity remained statistically stable for the other strains. The reduction of EA_24_ at 55 °C (although it was stable at higher temperature, i.e., to autoclaving), for example in the case of isolates L37, R43 and R55, could be ascribable to partial degradation of the bioemulsifiers as a result of the activation of extracellular hydrolytic enzymes (for example proteases and/or lipases) present in the culture supernatant of these isolates. Indeed, the cell free culture supernatants were pre-heated and further incubated at 55 °C once the organic solvents to be tested were added, which could has led the bioemulsifier polymeric compounds degradation. The activity of extracellular hydrolytic enzymes at relatively high temperature (55 °C) can be expected considering the origin of the isolates from a hot desert. On the contrary, a low temperature (4 °C) reduced EA_24_ of R55, R40 and N3 but did not affect that of the Tween 80 or the remaining strains (Fig. 2c).

The stability of the emulsification activity in hexadecane under stressful conditions was evaluated only for isolates exhibiting a significant stability in hexane and toluene solvents (L37, L45, L88, L97b, R4p and R55) as reported in Fig. 3a–c. The presence of different salt concentrations affected significantly the EA_24_ of strains L97b and R55 (Fig. 3a). Extreme pHs and temperature treatments significantly affected bacterial BEs performance in the case of L97b (pH 4), R55 (pH 4, 55 °C, autoclaving), R4p (55 °C, autoclaving), L37 and L88 (pH 12, 55 °C, autoclaving), up to complete EA_24_ loss in the case of strains L37, L88 and R4p at 55 °C and strains L88 and R4p after autoclaving (Fig. 3b, c).

Gutiérrez et al. [33] reported that heat (100 °C) and acid treatment (0.1 N) increased the relative activity of the bioemulsifiers produced by *Halomonas* sp. Activity increment was explained by a heating activation of polymeric emulsifiers releasing a higher number of emulsifying moieties from the biopolymers, enhancing the emulsifying capacity.

BEs not affected by salt stress (i.e. NaCl concentrations up to 300 g/L) and extremes of pH (2–13) have been previously described from *Paenibacillus* sp. #510 [30] as well as from marine *Bacillus* sp., *Halomonas* sp. and *Marinobacter* sp. [34]. Dubey et al. [35] reported that bioemulsifiers from *Pseudomonas aeruginosa* strain-PP2 and *Kocuria turfanesis* strain-J retained their activities at up to 20% NaCl and 121 °C. Marine *Rhodococcus* sp. has been reported to produce trehalolipid biosurfactant that formed emulsions stable to a wide range of temperatures (20–100 °C), pH (2–10) and NaCl concentrations (5–25%_w/v_) [36], and bioemulsifiers highly stable at low water activity and temperature extremes were reported from *Marinobacter* sp. [34]. EIs relatively stable under extreme pH, temperature, and salinity conditions have been previously reported from *Bacillus* spp. [23–25, 28].

The long-term stability of emulsions (reported as residual emulsification activity %) under standard screening conditions or after exposure to water, heat or extreme pH stresses was evaluated after a monitoring period of up to 30 months. The emulsions showed a high stability at room temperature, maintaining up to 100% of the initial EIs under standard conditions (Table 2), while different responses have been observed under the different stresses applied (Tables 3, 4, 5). Interestingly, some strains were able to retain unchanged activity in the presence of up to 15% NaCl (strain L45; Table 3), under acid/alkaline pHs (strains L45, L97b, R40 and R55; Table 4) and at 4 °C or after autoclaving (strains L45 and R55, respectively; Table 5).

**Table 2:** Residual emulsification activity (REA%) after an incubation period of 30 months under standard conditions

Isolate/surfactant	Toluene	Hexane	Hexadecane
Tween 80	94.74 ± 0.00	90.10 ± 9.58	16.67 ± 8.19
*Bacillus* sp. L37	95.24 ± 6.73	91.20 ± 4.19	0.00 ± 0.00
*Bacillus* sp. L45	91.71 ± 8.48	94.12 ± 4.71	7.56 ± 0.18
*Bacillus* sp. L97b	82.40 ± 3.95	94.76 ± 2.69	0.00 ± 0.00
*Bacillus* sp. L98	26.67 ± 1.33	58.93 ± 7.58	20.00 ± 1.00
*Bacillus* sp. R40	97.06 ± 4.16	84.62 ± 4.23	0.00 ± 0.00

**Table 3:** Residual emulsification activity (REA%) under low water activity after an incubation period of up to 30 months under standard conditions

Isolate/surfactant	Time (months)	8% NaCl			15% NaCl			30% NaCl
		Toluene	Hexane	Hexadecane	Toluene	Hexane	Hexadecane	Toluene
Tween 80	29	2.54 ± 3.60	59.74 ± 7.03	88.89 ± 4.44	0.00 ± 0.00	0.00 ± 0.00	0.00 ± 0.00	0.00 ± 0.00
*Bacillus* sp. L45	30	96.15 ± 3.26	100.00 ± 0.00	0.00 ± 0.00	95.32 ± 4.26	93.75 ± 0.00	0.00 ± 0.00	81.03 ± 7.79
*Paenibacillus tarimensis* L88	30	0.00 ± 0.00	0.00 ± 0.00	0.00 ± 0.00	11.93 ± 4.02	0.00 ± 0.00	0.00 ± 0.00	16.78 ± 1.98
*Bacillus* sp. L97b	29	66.52 ± 3.16	77.28 ± 6.88	0.00 ± 0.00	10.00 ± 0.50	12.92 ± 0.65	0.00 ± 0.00	0.00 ± 0.00
*Bacillus* sp. R40	29	64.91 ± 6.95	28.57 ± 1.43	nt	66.25 ± 8.84	na	nt	63.65 ± 7.52
*Bacillus* sp. R55	29	85.74 ± 5.54	46.67 ± 2.33	0.00 ± 0.00	91.70 ± 7.47	na	0.00 ± 0.00	87.94 ± 7.63

*nt* EI_24_ not tested, *na* not applicable (the stability was not monitored when no EI_24_ was recorded)

**Table 4:** Residual emulsification activity (REA%) at pH 4 and pH 12 after an incubation period of up to 30 months

Isolate/surfactant	Time (months)	pH 4			pH 12		
		Toluene	Hexane	Hexadecane	Toluene	Hexane	Hexadecane
Tween 80	29	96.22 ± 2.10	94.44 ± 0.00	89.47 ± 0.00	92.87 ± 2.23	96.22 ± 2.10	87.81 ± 1.15
*Bacillus* sp. L45	30	100.00 ± 0.00	77.09 ± 2.70	0.00 ± 0.00	66.43 ± 3.96	55.00 ± 7.07	0.00 ± 0.00
*Paenibacillus tarimensis* L88	30	82.26 ± 8.41	2.97 ± 4.20	0.00 ± 0.00	43.00 ± 8.44	27.38 ± 8.42	0.00 ± 0.00
*Bacillus* sp. L97b	29	52.04 ± 8.33	100.00 ± 0.00	0.00 ± 0.00	94.44 ± 4.72	98.30 ± 1.66	0.00 ± 0.00
*Bacillus* sp. L98	29	0.00 ± 0.00	na	nt	9.87 ± 1.75	54.25 ± 1.30	nt
Bacillus sp. N3	30	86.19 ± 0.67	0.00 ± 0.00	nt	26.30 ± 0.52	70.59 ± 3.53	nt
*Bacillus* sp. R40	29	0.00 ± 0.00	50.00 ± 0.00	nt	na	100.00 ± 0.00	nt
*Bacillus* sp. R55	29	100.00 ± 0.00	59.92 ± 2.81	0.00 ± 0.00	87.50 ± 4.38	100.00 ± 0.00	19.33 ± 0.97

*nt* EI_24_ not tested, *na* not applicable (the stability was not monitored when no EI_24_ was recorded)

**Table 5:** Residual emulsification activity (REA%) under different thermal treatments after an incubation period of up to 30 months

Isolate/surfactant	Time (months)	4 °C		55 °C			Autoclave treatment		
		Toluene	Hexane	Toluene	Hexane	Hexadecane	Toluene	Hexane	Hexadecane
Tween 80	29	91.27 ± 0.28	93.50 ± 1.33	0.00 ± 0.00	0.00 ± 0.00	0.00 ± 0.00	90.38 ± 1.29	77.78 ± 0.00	0.00 ± 0.00
*Bacillus* sp. L45	30	95.03 ± 5.14	100.00 ± 0.00	72.08 ± 0.92	56.41 ± 6.81	51.49 ± 8.00	66.79 ± 6.57	86.15 ± 8.70	0.00 ± 0.00
*Paenibacillus tarimensis* L88	30	62.50 ± 8.84	0.00 ± 0.00	0.00 ± 0.00	na	na	na	na	na
*Bacillus* sp. L97b	29	92.26 ± 1.52	92.72 ± 5.42	75.00 ± 3.75	90.42 ± 0.00	0.00 ± 0.00	28.57 ± 0.00	81.25 ± 4.06	0.00 ± 0.00
*Bacillus* sp. L98	29	79.41 ± 8.32	83.29 ± 4.13	0.00 ± 0.00	0.00 ± 0.00	nt	0.00 ± 0.00	79.48 ± 6.33	nt
*Bacillus* sp. R4p	29	92.08 ± 2.37	77.05 ± 2.90	0.00 ± 0.00	0.00 ± 0.00	na	0.00 ± 0.00	0.00 ± 0.00	na
*Bacillus* sp. R55	29	83.95 ± 8.72	68.40 ± 8.86	79.40 ± 8.94	77.59 ± 3.88	88.67 ± 4.43	97.94 ± 1.97	100.00 ± 0.00	0.00 ± 0.00

*nt* EI_24_ not tested, *na* not applicable (the stability was not monitored when no EI_24_ was recorded)

Long-term EI stabilities have been previously observed for the bioemulsifiers produced by *Pedobacter* sp. strain MCC-Z where the emulsions remained stable for 4 months [37]. Extended stability for longer incubation times (18–30 months) under low water activity or after thermal treatments was also observed for bioemulsifiers produced by *Marinobacter* sp. isolates [34], while no reports on long period incubation under pH extremes are available to the best of our knowledge.

### Water retention and humidity uptake by a BS/BE-treated sandy soil

Based on the characterization of the surface activity of the produced bacterial compounds two isolates (L45 and R43) were selected for further tests. While L45 produced only BEs, R43 was able to produce BEs that significantly reduced IFT but exhibited low emulsification activity. Their BS/BEs were used for testing their ability to improve water content and humidity uptake of sandy soil. An additional strain, V3E1 isolated from arid agricultural soil, was also used. The isolate V3E1 was included in the study since it has been shown to produce extracellular polymeric substances (EPS), and based on literature reports EPS have been found to play an important role in water retention in biological soil crusts [8]. The isolate produced surface-active compounds that were able to reduce the surface tension of the cultivation medium up to 69.81 ± 0.88 mN/m and to emulsify the different solvents used exhibiting EI_24_ (%) of 55.93 ± 5.76; 5.00 ± 2.36 and 11.67 ± 2.36 for toluene, hexane and hexadecane, respectively.

**Fig. 4:**
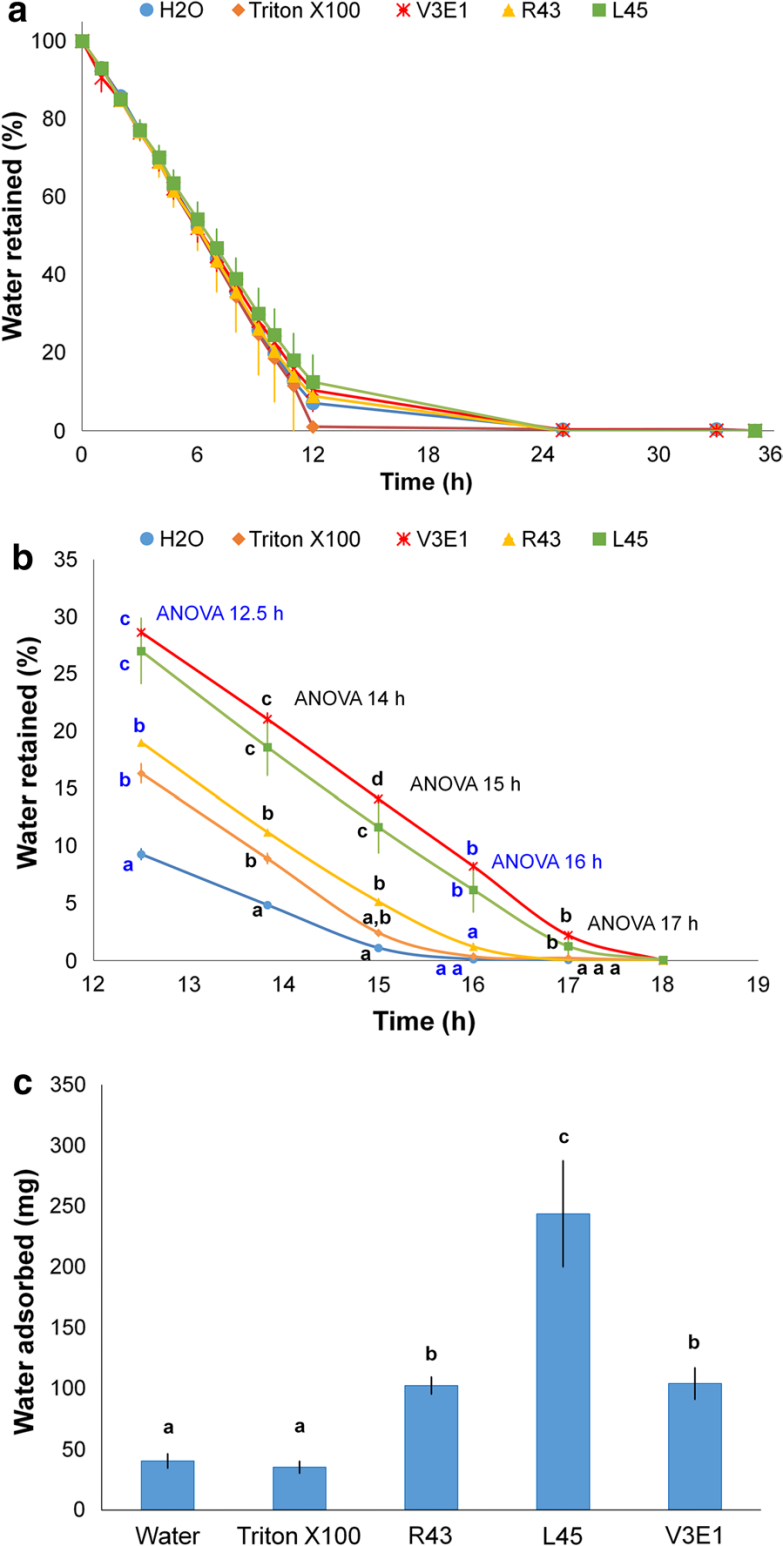
Results of water retention and moisture uptake assays by a sandy soil treated with a BS/BE solution, Triton X-100 or tap water. **a** Trend of water content during the first cycle of irrigation; **b** results of water retention assay recorded after 12.5 h of the onset of the second irrigation. Analyses of variance performed on water content after 12.5, 14, 15, 16 and 17 h (ANOVA 12.5 h to ANOVA 17 h), showing statistically significant differences among the samples (ANOVA 12.5 h: p < 0.0001, F = 139.41, df = 4; ANOVA 14 h: p < 0.0001, F = 138.19, df = 4; ANOVA 15 h: p < 0.0001, F = 127.16, df = 4; ANOVA 16 h: p < 0.0001, F = 72.06, df = 4; ANOVA 17 h: p < 0.0001, F = 17.80, df = 4; ANOVA 18 h: p = 0.15, F = 2.00, df = 4). According to post–hoc analysis (α = 0.05), means sharing the same letter are not significantly different from each other; **c** results of moisture uptake assay. ANOVA showing statistically significant differences among the samples (p < 0.0001; F = 148.70; df = 4). According to post–hoc analysis (α = 0.05), means sharing the same letter are not significantly different from each other

The water retention capacity of sandy soils treated with BS/BE solutions was monitored after two irrigation events using sterilized tap water. No significant differences between the treatments were observed during the first ten hours of monitoring, after the start of the first irrigation (Fig. 4a). Based on this, a second irrigation treatment was performed and new measurements were carried out (Fig. 4b). Statistical analyses showed that after 12.5 h, compared to control (not subjected to BS/BE treatment) which retained only 9.1 ± 0.9% (Fig. 4b), Triton X-100, R43, L45 and V3E1-treated sandy soil samples significantly reduced water evaporation retaining 16.3 ± 0.8; 19.0 ± 0.1; 27.0 ± 3.5 and 28.6 ± 1.2% of the initial water, respectively. Hence, comparing the data obtained we can show that after 12 h bacterial BS/BE significantly increased water retention, with values up to 314.3% (V3E1) higher than the untreated control. This capability to retain a significantly higher water amount, compared to control, was statistically confirmed also during the two following hours. However, after 16 h, samples irrigated with Triton X-100, R43 and water were found to loose almost completely their water content (Fig. 4). Those irrigated with L45 or V3E1 BS/BE still retained a significantly higher amount (6.2 ± 2.4 and 8.2 ± 0.0%, respectively) of the initial water, confirming the BS/BE capacity to improve water retention in sandy soil. All the samples lost their water content after 18 h from the start of the second irrigation (Fig. 4b). It is of note that the sandy soil samples treated with BE produced by L45 and V3E1, which did not reduce IFT, retained higher water content compared to those treated with Triton X-100 or BS from isolate R43, which instead was able to reduce the IFT. These results suggest that L45 and V3E1 produce high molecular weight polymers with bioemulsification activity that can improve sandy soil compactness and/or absorb high water amounts, finally determining higher water retention and reduction of evaporation rates. Improvement of the water retention capacity was observed in induced biological soil crusts as a function of their total carbohydrate content [8].

The capability of BS/BE-treated sandy soil to absorb humidity from the surrounding environment was also evaluated after 36 h of incubation at 30 °C and high relative humidity. Samples treated with tap water or Triton X-100 absorbed the same amounts of humidity, which was statistically different from the quantity of water vapor absorbed by the sand samples treated with L45, R43 and V3E1. The BS/BE produced by these bacteria significantly improved the amount of water absorbed by the sandy soil. Data reported in Fig. 4c showed that L45-treated sand sample was able to absorb the highest amount of water vapor, i.e. 607.7% higher than the control treated with tap water. Similarly, V3E1 and R43 BS/BE determined an uptake of humidity 259.1 and 254.9%, respectively, higher than control (Fig. 4c). Interestingly, the strain L45, that showed the highest humidity uptake rate, exhibited also the ability to highly emulsify different solvents (Table 1).

The effect of the bacterial BS/BE on the soil water retention and humidity absorption highlights the potential of bacterial strains typical of arid environments to improve the hydrological properties of sandy soils, even though we must consider that these results were obtained with a single soil type. Different soils may present different responses to bacterial BS/BE and a further research is needed to improve the knowledges regarding the response of soils to the use of surfactants for improving its hydrological properties.

## Conclusions

We showed that bacteria from arid environments can produce polyextremotolerant bioemulsifiers that are functional in broad ranges of pH and temperature and in the presence of 30%_w/v_ NaCl. The emulsions were stable up to 30 months incubation under several conditions. The partially purified BS/BE produced by isolates L45, R43 and V3E1 significantly improved water retention and humidity uptake of a sandy soil compared to Triton X-100 or tap water. The data offer insights into the biotechnological properties of BS/BE from bacteria inhabiting non-conventional environments and their potential role for environmental protection and the improvement of soil hydrological properties in arid regions. Indeed, the use of BS/BE as additives in the irrigation water would be an interesting approach to promote plant stress to drought as an alternative to the conventional inoculation of living microbial cells, overcoming potential limitations related to over competition of the inoculated bacteria by the rhizosphere microbioma. However, the application of BS/BE should be carefully evaluated taking into consideration the characteristics of the soil to be irrigated since BS/BE–soil interaction can vary, resulting in a decrease or an increase of the soil hydrophobicity.

## Additional files

**Additional file 1:Table S1.** List of the 23 bacterial strains used in this work.

**Additional file 2: Figure S1.** Phylogenetic affiliation of partial 16S rRNA gene of the 23 bacterial isolates obtained from chott and desert in the south of Tunisia constructed using MEGA6 package. Neighbor-Joining phylogenetic tree was built using MEGA 6, computing the evolutionary distances using the Kimura 2-parameter model.
